# Increasing the Impact Strength of Grass Fiber Polypropylene Composites with Virgin and Recycled Polypropylene

**DOI:** 10.3390/polym18202540

**Published:** 2026-10-16

**Authors:** Katharina Grünheide, Eric Stuckert, Heiko Hofmann, Martin Moneke

**Affiliations:** Department of Mechanical and Plastics Engineering, Darmstadt University of Applied Sciences, 64295 Darmstadt, Germany; katharina.gruenheide@biovox.systems (K.G.);

**Keywords:** natural fibers, permanent pasture grasslands, polypropylene, compounding, coupling agents, adhesion, impact strength, melt flow index, recycling

## Abstract

Natural fibers from different plants are widely used as reinforcing fillers in polymers due to their ecological, economic and technical benefits, with applications in all major industries. Since a typical drawback of incorporating natural fibers into polymers is a reduction in impact strength, this work investigates ways to enhance impact strength. Grass fibers of 30 wt. % from permanent pasture grasslands are compounded with polypropylene and varying amounts of polypropylene-graft-maleic anhydride with three different degrees of grafting as coupling agents and ethylene propylene diene rubber as the impact modifier. The impact strength and the melt flow index are measured for each compound while the adhesion between the fibers and matrix is investigated by scanning electron microscopy. It can be shown that around 5 wt. % of coupling agents are sufficient to increase impact strength to a saturation level, while the impact modifier has no significant influence. Through multiple extrusion cycles of a pure grass fiber polypropylene compound, it can be shown that the melt flow index increases constantly with the number of extrusion cycles, while the impact strength remains constant after an initial increase, indicating matrix degradation but enhanced fiber matrix coupling.

## 1. Introduction

A number of reasons to use natural fibers as reinforcement in polymer composites have been reported including ecological benefits (reduced carbon footprint [1,2], lower energy consumption for fiber production compared to, e.g., glass fibers [1,3], use of biobased materials [1,4]), economic benefits (accessibility [4], lower material costs [1,2,3,4,5,6,7], reduced price fluctuation) and technical benefits (lower density [1,2,4] enabling lightweight structures, acoustic damping [1] and energy absorption, reduced brittleness [1] and splintering and hence favorable crash behavior [2,7]).

As a result, there are many applications for natural fiber composites in almost every industry. Applications in the automotive industry include dashboards, door panels, headliners, seat backs, trunk trims, and engine encapsulation [8,9]. First mentions date back to 1942 when Henry Ford presented a prototype car with hemp fibers and to the 1950s with the Trabant car body made from epoxy resin and cotton fibers [4]. In the construction and building industry, mainly wood fibers are used for decking, window and door profiles, fences, railings and roof tiles [10,11], while in the packaging industry sustainable packaging based on natural fibers and bio-based or biodegradable polymers is of particular interest [12,13,14]. Applications are also found in the aerospace industry [7,14,15], in consumer goods and furniture [13,16], in defense and military applications [17,18], and biomedical applications [14,19].

When looking more specifically at the technical benefits, first of all, the tensile strength and tensile modulus of single natural fibers are lower than that of E-glass-fibers [2,20]. Hence, the addition of natural fibers increases the tensile modulus as well as the tensile and bending strength of a polymer but to a lesser extent than the addition of glass fibers [21]. However, the increase in these properties strongly depends on the quality of the fiber–matrix interface [6,22,23]. Therefore, coupling agents are a means to increase impact strength by reducing interfaces and thus preventing or minimizing crack propagation.

Technical drawbacks that come with the incorporation of natural fibers compared to synthetic fibers are lower impact strength [1,5,8,10,23,24,25,26,27], poor compatibility with hydrophobic polymers [6,28,29], higher moisture absorption. [2,19,30], and processing consistency when using a material with large variability due to its natural origin.

Impact modifiers like ethylene propylene diene rubber are a means to control impact strength. They are also used with natural fibers [4,10,31,32,33] but their effects can be complex [8]. Therefore, a hypothesis is that modification of the matrix only helps to a limited extent if the interfaces between fibers and the matrix remain unchanged, as cracks may spread unhindered through the interfaces.

An additional technical challenge is that the variability of natural fibers poses a challenge for the reproducibility of the properties of the resulting compounds [1]. This is especially true for the mechanical properties, which are most relevant for technical products for which fiber-reinforced plastics typically are being used [34].

In general, in natural fiber polymer composites, the matrix material can be a thermoset or a thermoplastic, with the latter being ecologically more favorable due to the better recyclability. Thermoplastics being studied extensively are polypropylene, polyethylene, polystyrene and polyvinyl chloride [5,10]. Among thermoplastics, polypropylene is increasingly used for technical applications and hence is very interesting in combination with natural fibers as reinforcement.

Natural fibers can be extracted from a variety of plants and are typically categorized according to the origin of the plant, i.e., bast, leaf, seed, fruit, wood [10], or grass [2,5,10]. Major fiber sources worldwide are sugar cane, bamboo, jute, kenaf, flax, grass (American, Asian and African varieties such as big bluestem, snake grass, bamboo, esparto, and sabai), sisal and hemp [5,35], while in Europe regional natural fiber sources are hemp [5], flax, wood while jute, kenaf, and sisal, imported for production. All of these plants are typically grown in monocultures. However, there is also mention of fibers derived from grass from European grasslands [36]. This report describes the manufacture and use of grass fibers and byproducts from agricultural grasslands for different applications, highlighting insulation and construction panels, gardening supplies, geo textiles and polymeric composites. In line with these findings, the German company BioWert produces a plastic compound with specially cultivated German ryegrass as a natural reinforcing fiber. The German ryegrass is derived from monocultures, which thereby reduces biodiversity. To increase biodiversity, conversely, an approach would be to extract fibers from all plants growing on the same piece of land. Due to the ease of harvesting, this is a readily accessible route for permanent pasture grasslands in Europe.

To close gaps for European grass, this paper therefore describes the development of a compound based on polypropylene and regionally grown permanent pasture grass with the addition of an impact modifier and coupling agents, with the goal of increasing impact strength.

## 2. Materials and Methods

The injection grade polypropylene homopolymer was supplied by TotalEnergies (Brussels, Belgium), grass fibers from permanent pasture grasslands by Biowert Industrie GmbH (Brensbach, Germany), coupling agents by Polymer Asia (C1) (Ho Chi Minh City, Vietnam) and BYK (C2 + C3) (Wesel, Germany), and the impact modifier (IM) by Melos GmbH (Melle, Germany) (Table 1). Regrinding of injection-molded articles from the virgin polypropylene used here was performed at Cortec GmbH (Wald-Michelbach, Germany).

The rheological properties of the polypropylene homopolymer and the coupling agents differ significantly, as can be seen from Table 2, keeping in mind that the data taken from the suppliers’ data sheets are measured under different conditions and to either ISO or ASTM standards. Still, it is apparent that all coupling agents show a substantially lower viscosity than the polypropylene used here, while the viscosity of C1 is lower than that of C2, which is lower than that of C3.

**Figure 1:**
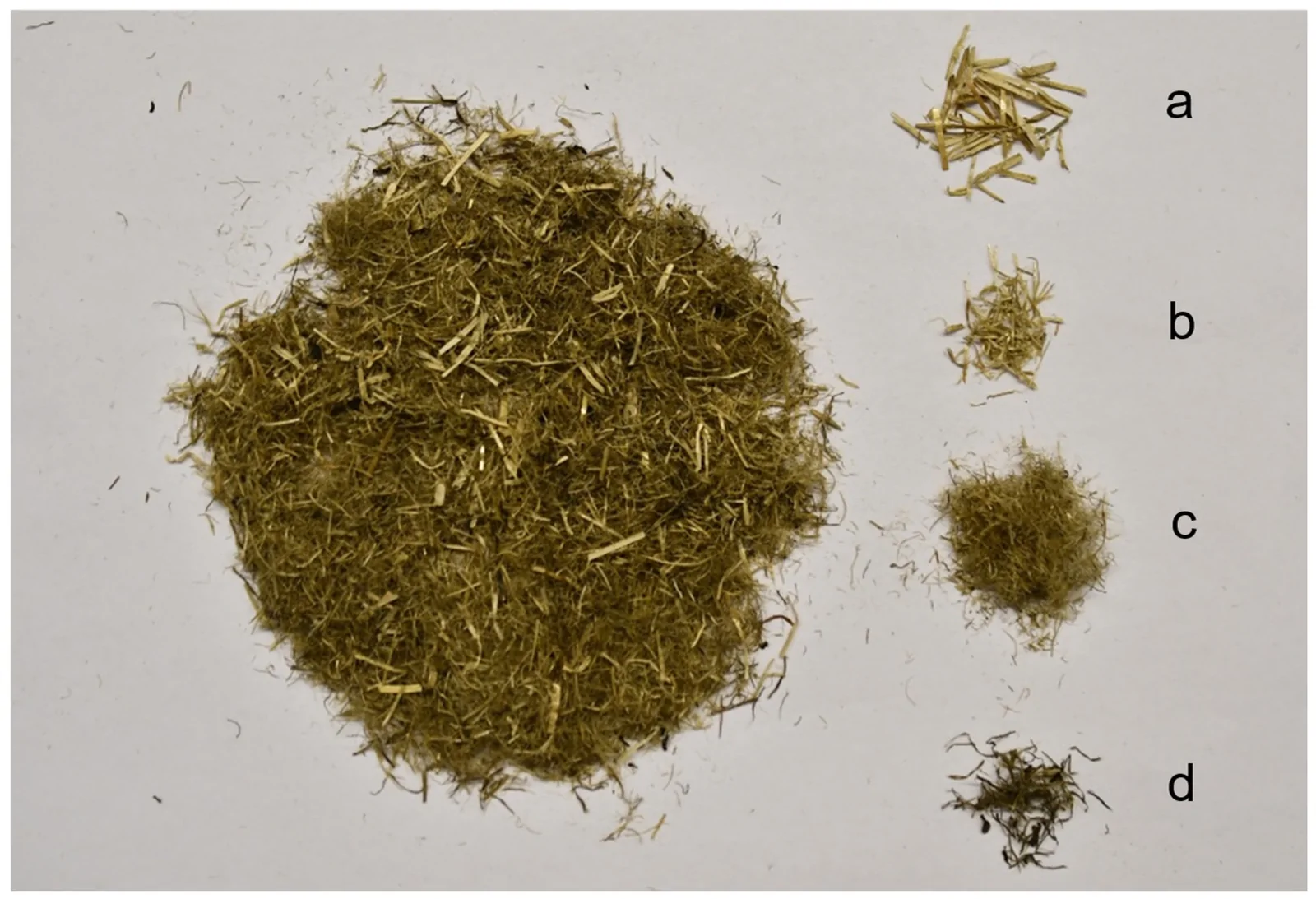
Fermented, washed, dried and cut permanent pasture grassland grass from the Vogelsberg region, Germany; (**left**) fiber mixture; (**right**) fibers separated into four clusters ((**a**) coarse yellow fibers; (**b**) fine yellow fibers; (**c**) fine green fibers; (**d**) black fibers and particles).

The grass fibers harvested in the Vogelsberg region of Germany undergo several processing steps at Biowert Industrie GmbH, including anaerobic lactic acid fermentation (silage), washing, mechanical grinding, drying, and cutting. Cutting is performed using a commercial cutting mill to retrieve fibers of a length of approximately 20 mm. The resulting fibers are bundles of cellulose fibers bonded by lignin and hemicellulose which clearly resemble the original grass stems and leaves in form and structure. A first analysis is performed visually by classifying the fibers into four distinct clusters based on their outer appearance (see Figure 1).

**Figure 2:**
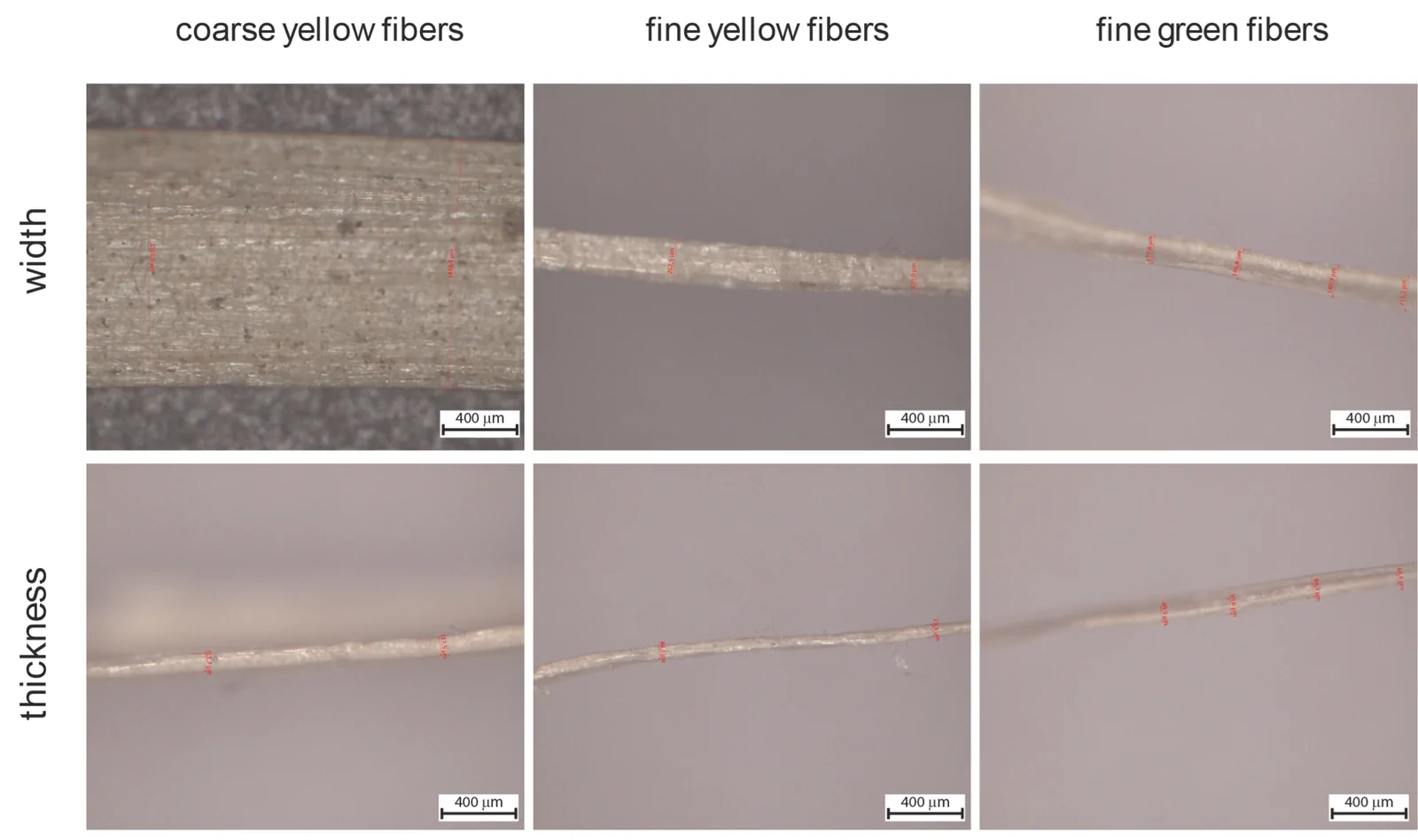
Microscopic images of grass fibers from the three clusters “coarse yellow fibers”, “fine yellow fibers”, “fine green fibers” looking at the wide and narrow surface of fibers with rectangular cross-sections.

The grass fibers exhibit rectangular cross-sections with different sizes, as shown in Figure 2 and Table 3. The coarse yellow fibers are flat with a width-to-thickness ratio of 13.3. The fine yellow fibers and the fine green fibers still have a rectangular cross-section, however less pronounced, with width-to-thickness ratios of 2.5 and 1.7, respectively. The black fibers and particles have not been measured due to their large diversity ranging from fractured fibers to debris and even remains of insects. The mean value and the standard deviation are calculated from four measurements along five fibers per cluster. The large variation in sizes even along one fiber and more so among different fibers of one cluster is expressed by the large standard deviation.

For the compounds identified in Table 4, all grass fibers from the same batch are used as delivered and without classification, i.e., what is shown in Figure 1 is used.

The compound consists of 30 wt. % grass fibers, 60–70 wt. % polypropylene (PP), and 0–10 wt. % coupling agents and impact modifier, as shown in Table 4.

Both, virgin PP material and PP regrind were investigated to evaluate potential differences in processing behavior and material performance. To assess the compatibility and dispersion efficiency within the heterogeneous grass fiber system, three different coupling agents C1 to C3 were used at different concentrations in order to systematically investigate their influence on interfacial adhesion and overall composite properties.

**Figure 3:**
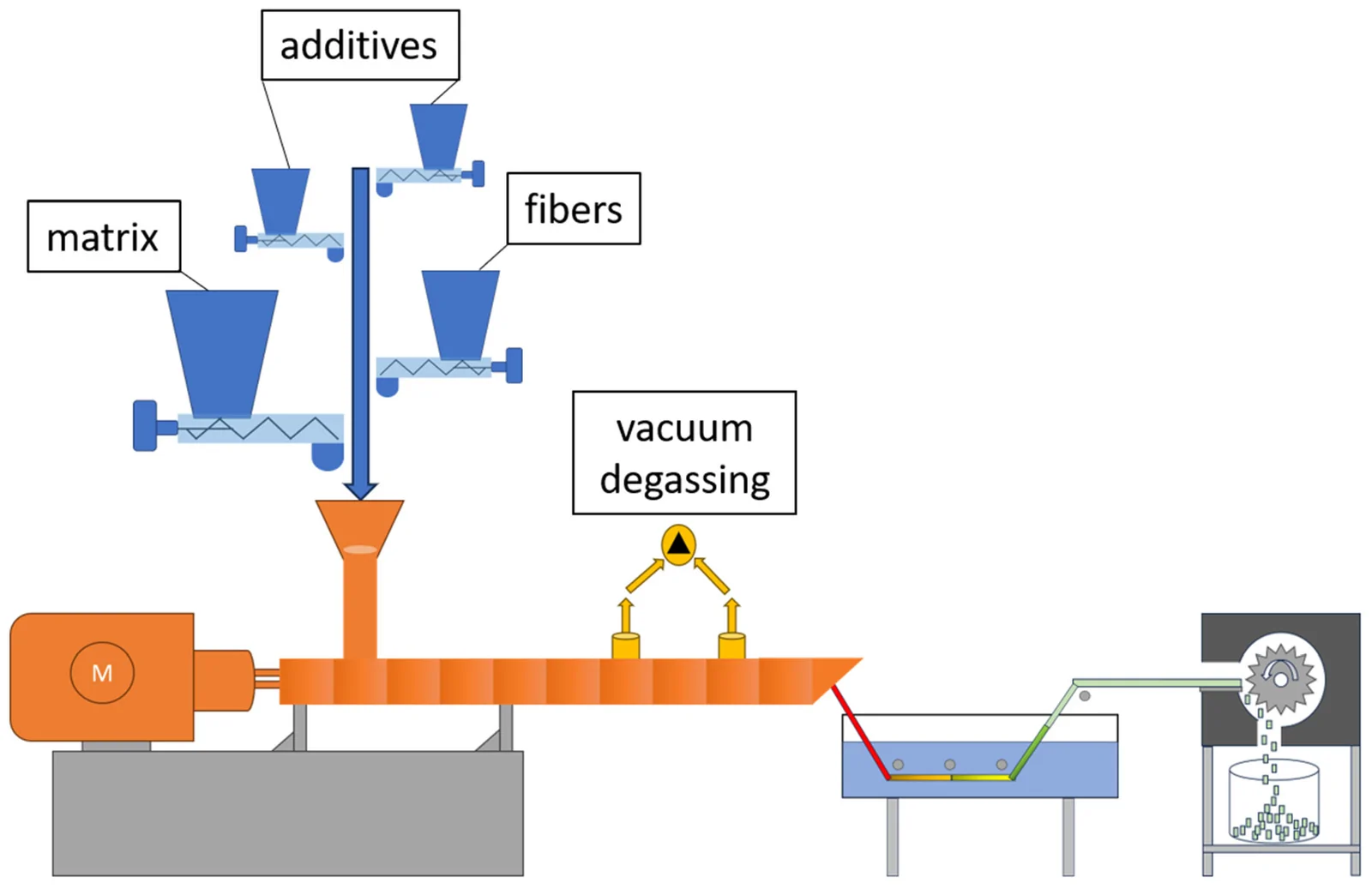
Setup of compounding line showing feeding units, vacuum degassing, cooling bath, and pelletizing unit.

Compounding of the grass fiber polypropylene composites was performed with a twin-screw extruder, gravimetric dosing units, vacuum degassing, water cooling and pelletizing, as shown in Figure 3.

**Figure 4:**
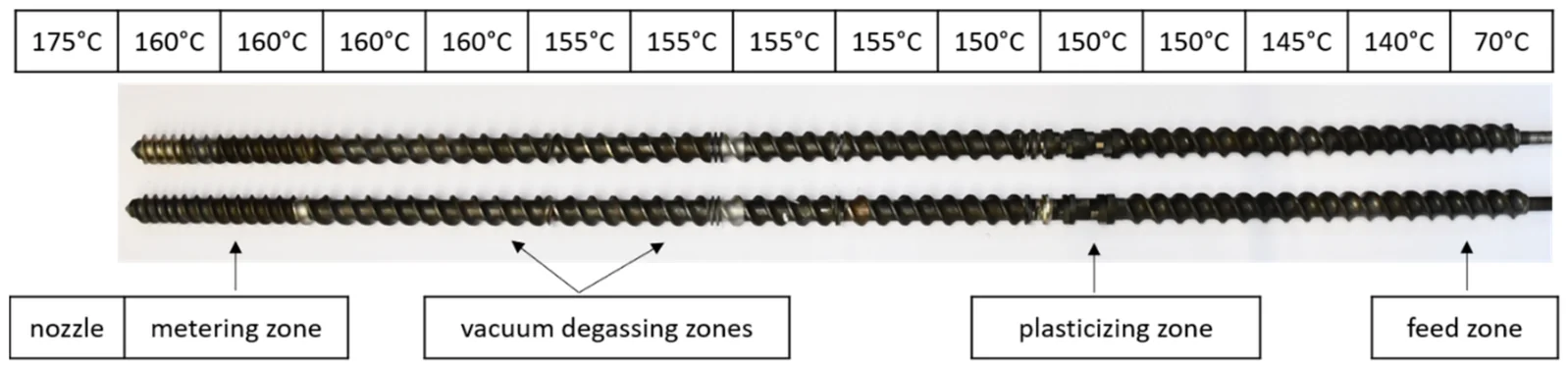
Screw configuration and temperature profile for co-rotating twin-screw extruder with feeding zone, plasticizing zone, two vacuum degassing zones, and metering zone.

All components are fed into the extruder via the main hopper. Some of the moisture on and within the grass fibers (7–9 wt. % on delivery) evaporates immediately upon entry into the warm feed zone (barrel temperature of 70 °C, see Figure 4) as steam is observed leaving the feed zone. The remaining humidity will eventually evaporate from the grass within the extruder and serves as a stripping agent [37] for volatile organic compounds (VOCs) which result from the decomposition of grass at higher temperatures (more than 140 °C barrel temperature downstream of the hopper). These gases are extracted from the compounding line via vacuum degassing. Stripping agents are also typically used in mechanical recycling of post-consumer plastic waste to extract VOCs from the compound in the degassing zones on an extrusion line [38]. Adding the fibers via the main hopper also allows for higher throughputs due to the larger opening in the feed zone compared to side feeding.

The extruder is a co-rotating twin-screw extruder ZSK 30 (Coperion GmbH, Stuttgart, Germany). The screws have a diameter of 30 mm and a length of 1350 mm, yielding an L/D ratio of 45. The screw profile was developed in a previous project for flax fibers and is shown in Figure 4. In the plasticizing zone, two blocks of kneading discs with stagger angles of 45° followed by two blocks of thinner kneading discs with stagger angles of 45° are used for plasticizing. Prior to the degassing zones are reverse-conveying screw elements.

Also shown in Figure 4 is the temperature profile along the barrel used for compounding the grass fiber polypropylene composites. The screw speed is 320 rpm, resulting in a throughput of 4.5 kg/h.

Gravimetric feeding units (Kubota Brabender Technologie GmbH, Duisburg, Germany) are utilized to ensure precise feeding, which is crucial at the low additive mass flow rate given by the throughput of 4.5 kg/h. Especially for fiber feeding, gravimetric feeding compensates fluctuating bulk densities and fiber agglomeration. Also, various modifications are made to the fiber feeding unit including a hopper with straight walls, spiral screws with optimized diameter, pitch, and flight width, and exhaust pipes with inner profiles.

To examine the possible degradation behavior of the compounds during processing and the effects of multiple mixing, the material is processed up to six times using the same equipment as for compounding with a single dosing unit only and the parameters described in above for the compounding process.

The compounds are injection-molded into type A1 tensile test specimens according to ISO 527-2 and ISO 20753 using an Arburg 470 E injection molding machine (ARBURG GmbH + Co KG, Loßburg, Germany). The injection molding parameters are shown in Table 5. Before injection molding, the compounds are dried at 80 °C for at least 4 h in a circulating air oven to a moisture content of 0.18 wt. %.

From the test specimens, the center pieces (80 mm × 10 mm × 4 mm) are milled for impact testing. Specimens and granulate are stored under standard atmospheric conditions for conditioning and testing according to ISO 291 (23 °C; 50% relative humidity) for at least 48 h.

Charpy impact tests are performed according to ISO 179-1 using a pendulum impact tester (ZwickRoell B5113.300, ZwickRoell GmbH & Co. KG, Ulm, Germany) with a 2 J pendulum and unnotched specimens. The mean value and standard deviation are calculated from five measurements.

The front surfaces of granules which are cut during the pelletizing process are analyzed by SEM (Hitachi SU5000, Hitachi Europe Limited, Slough, UK) after being coated with a 20 nm thick layer of gold employing the high-vacuum mode with an acceleration voltage of 5.0 kV and spot intensity of 30%. Using granules directly after compounding eliminates influences of injection molding and breaking specimens to produce fracture surfaces.

## 3. Results and Discussion

Initial material tests for compounds made from 30 wt. % grass fibers with virgin polypropylene (compound P0, see Table 4) and polypropylene regrind (compound P0r, see Table 4), respectively, showed no significant differences in mechanical properties, as shown in Table 6. Only the standard deviation for the impact strength was higher when using regrind. Therefore, both matrix polymers were used for further investigation with a focus on virgin polymers.

**Figure 5:**
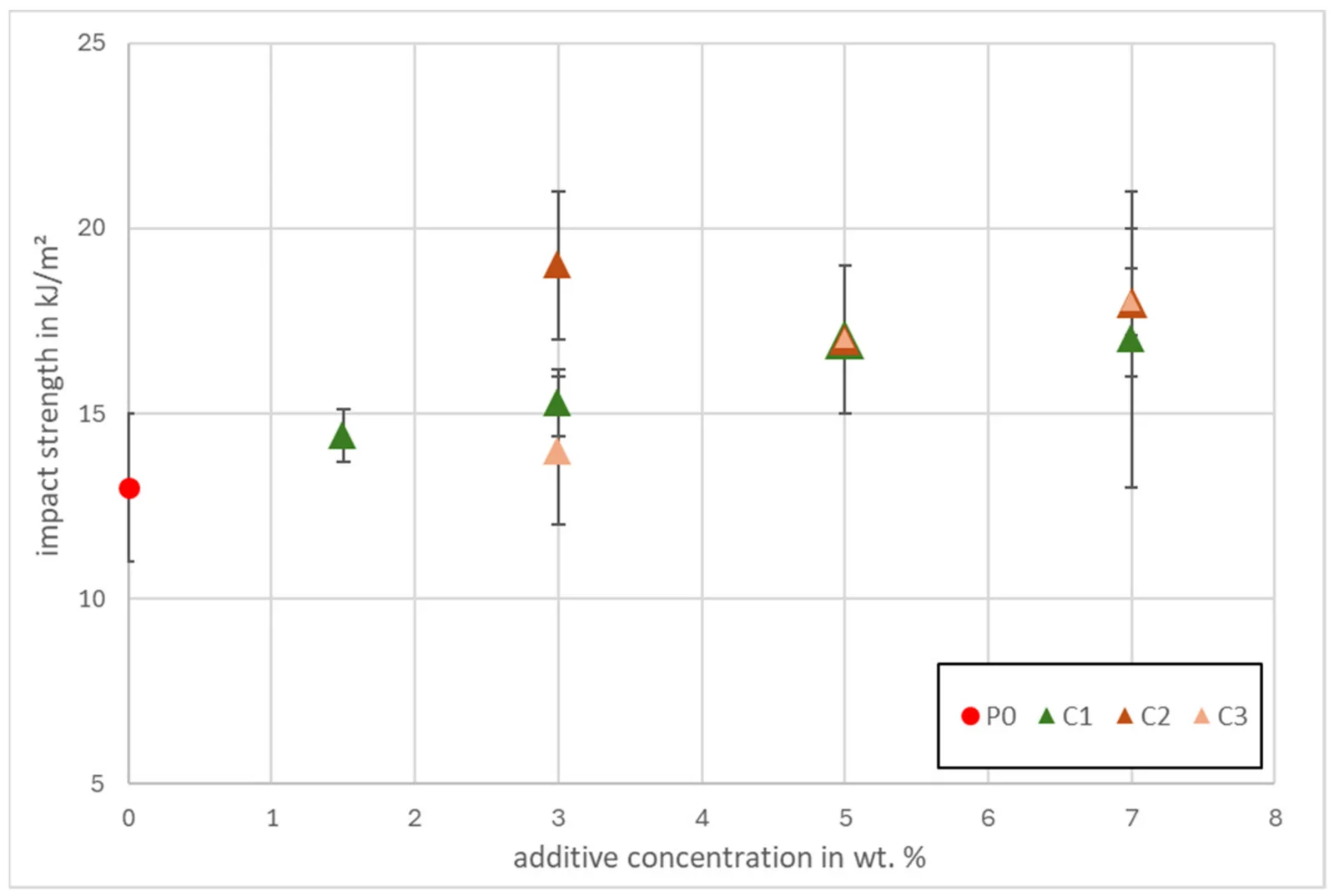
Impact strength of compounds P0 to P10 with 30 wt. % grass fibers and various amounts of three coupling agents with different degrees of grafting (C1: 1.0%, C2: 1.4%, C3: 2.0%).

Impact strength measurements generally showed an increase in impact strength with increasing concentration of the coupling agent, as expected. However, the effect of the three different coupling agents on the impact strength of the compounds is more complex, as shown in Figure 5. While compound P0 has an impact strength of 13 kJ/m^2^, coupling agent C1 leads to the typical increase in impact strength up to 17 kJ/m^2^ with increasing concentration from 1.5% to 5%, yielding a constant level of 17 kJ/m^2^ for concentrations higher than 5%.

Compounds with coupling agent C2 with a degree of grafting of 1.4% exhibit the highest impact strength at a concentration of 3%, with values fluctuating on the same level for higher concentrations, indicating that the threshold concentration to reach saturation might be below 3%.

Not in line with these findings is that coupling agent C3, with the highest degree of grafting, namely 2.0%, again shows an increase in impact strength with increasing concentration of the coupling agent, very similar to the effects of C1.

This unusual finding might be attributed to the use of different batches of grass fibers, as a review revealed that the grass fiber batches used were manufactured at different times; so, the influence of varying environmental conditions cannot be ruled out. This again highlights the overruling influence of grass fiber quality on compound characteristics and the fact that different degrees of grafting of the MA-g-PP coupling agents do not have the largest influence on impact strength.

**Figure 6:**
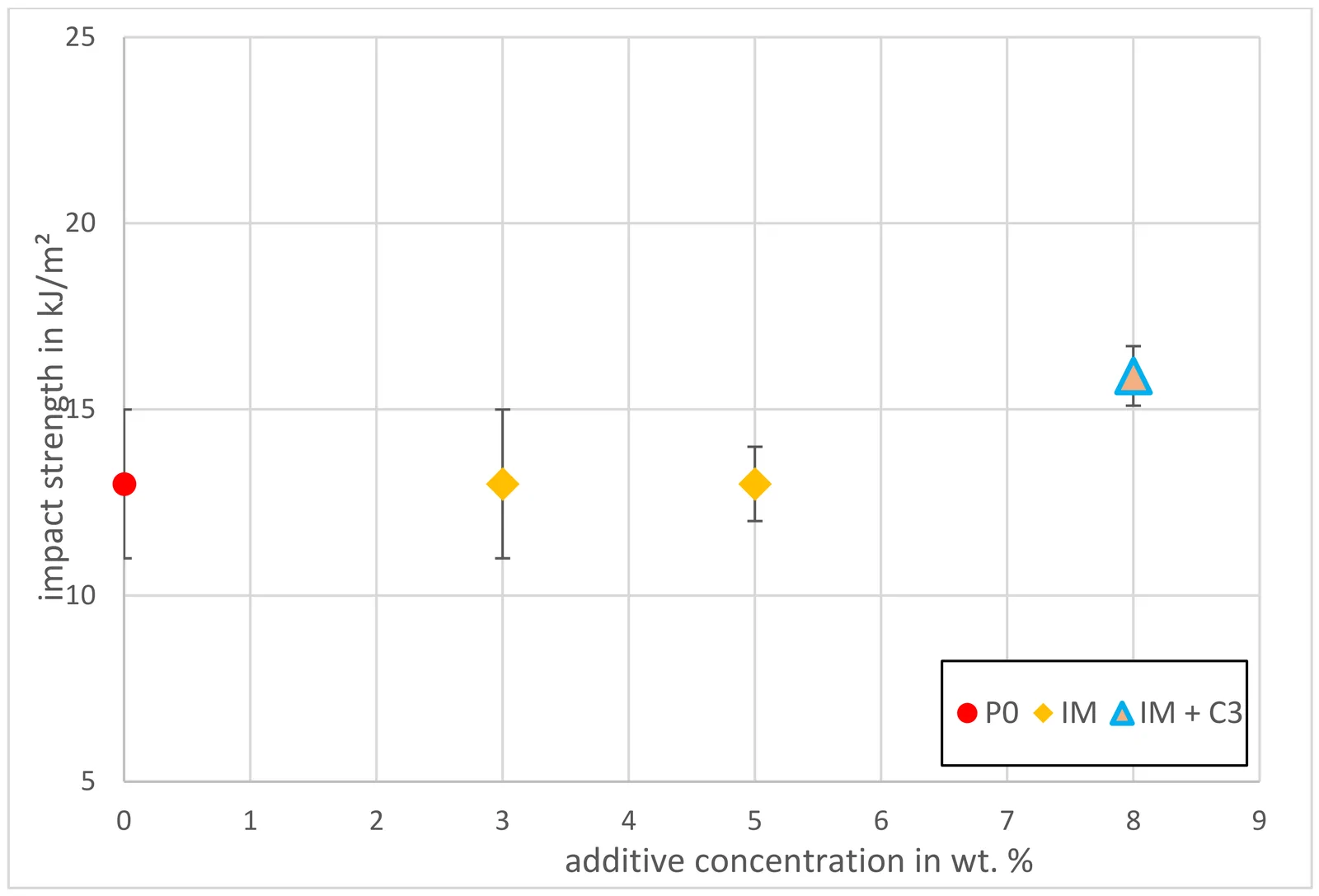
Impact strength of compounds P0 and P11 to P13 (regrind) with 30 wt. % grass fibers and various amounts of impact modifier and coupling agent (P13 with IM + C3: 3% IM + 5% C3).

On addition of 3% or 5% of the impact modifier to compound P0, there was no change in impact strength, which remained at 13 kJ/m^2^, as shown in Figure 6. An increase in impact strength to 16 kJ/m^2^ can only be observed when 5% of coupling agent C3 was added in combination with the impact modifier. However, this value is lower than an impact strength of 17 kJ/m^2^ when coupling agent C3 was added at a concentration of 5% alone, as shown in Figure 5. The observation that for grass fibers in polypropylene, coupling agents have a larger effect on impact strength than impact modifiers leads to the conclusion that the interface between fibers and the matrix serves as the predominant factor influencing mechanical properties, which is in line with findings for hemp in polypropylene [22,23].

**Figure 7:**
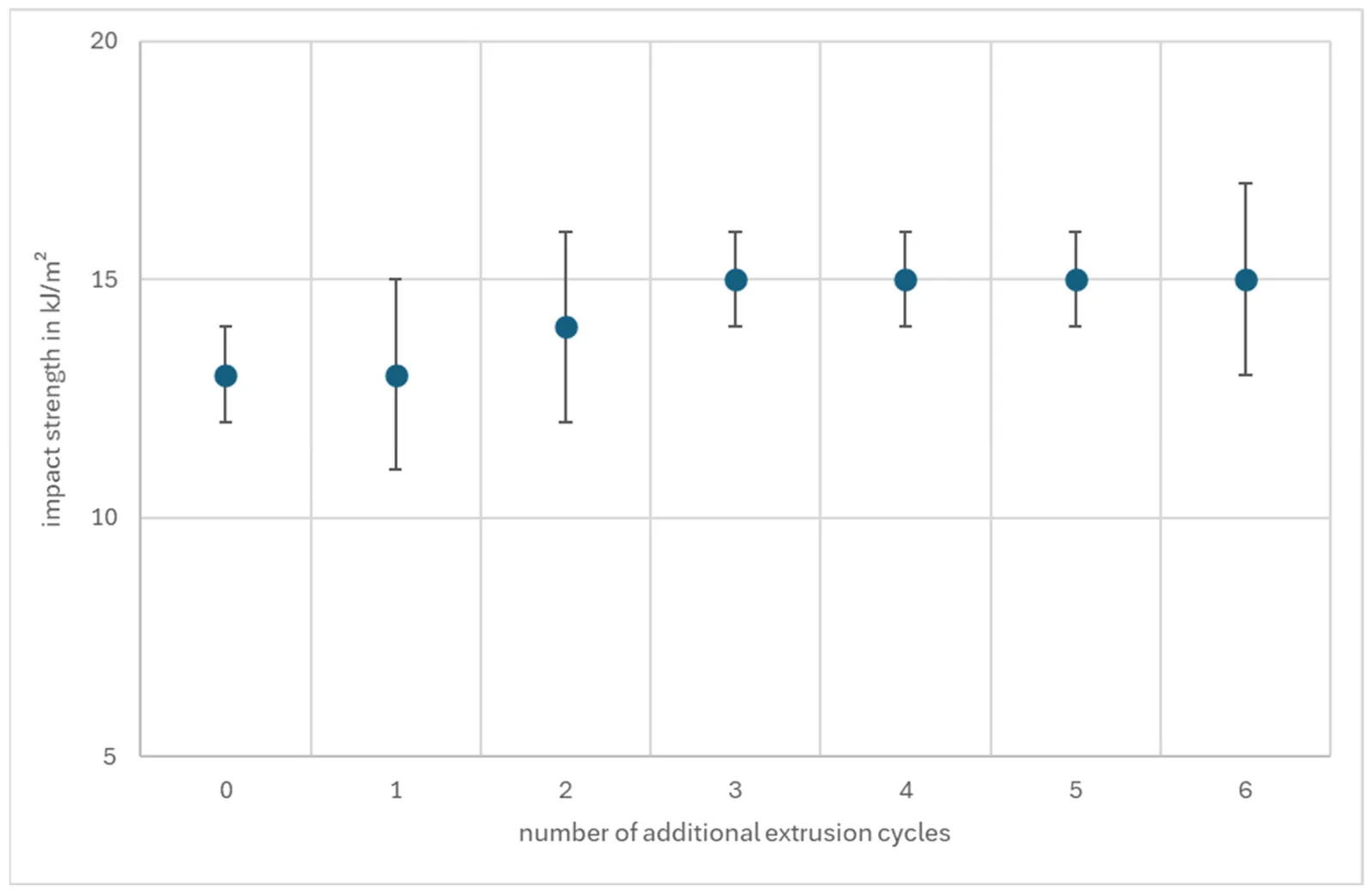
Impact strength of compound P0 versus number of additional extrusion cycles.

To further prove that the matrix does not significantly contribute to the impact strength of the compound, specimens of compound P0 were tested after multiple extrusion cycles, as shown in Figure 7. During multiple extrusion cycles, the following trend can be observed: starting from the initial value of 13 kJ/m^2^ for compound P0, the impact strength increased to 15 kJ/m^2^ within the first three extrusion cycles and then remained at this level. This might be attributed to better mixing of the fibers, coupling agent and matrix until the best mixing was reached. However, multiple processing may simultaneously cause fiber shortening, changes in fiber length distribution, and degradation of lignocellulosic components, which may also influence the impact strength.

**Figure 8:**
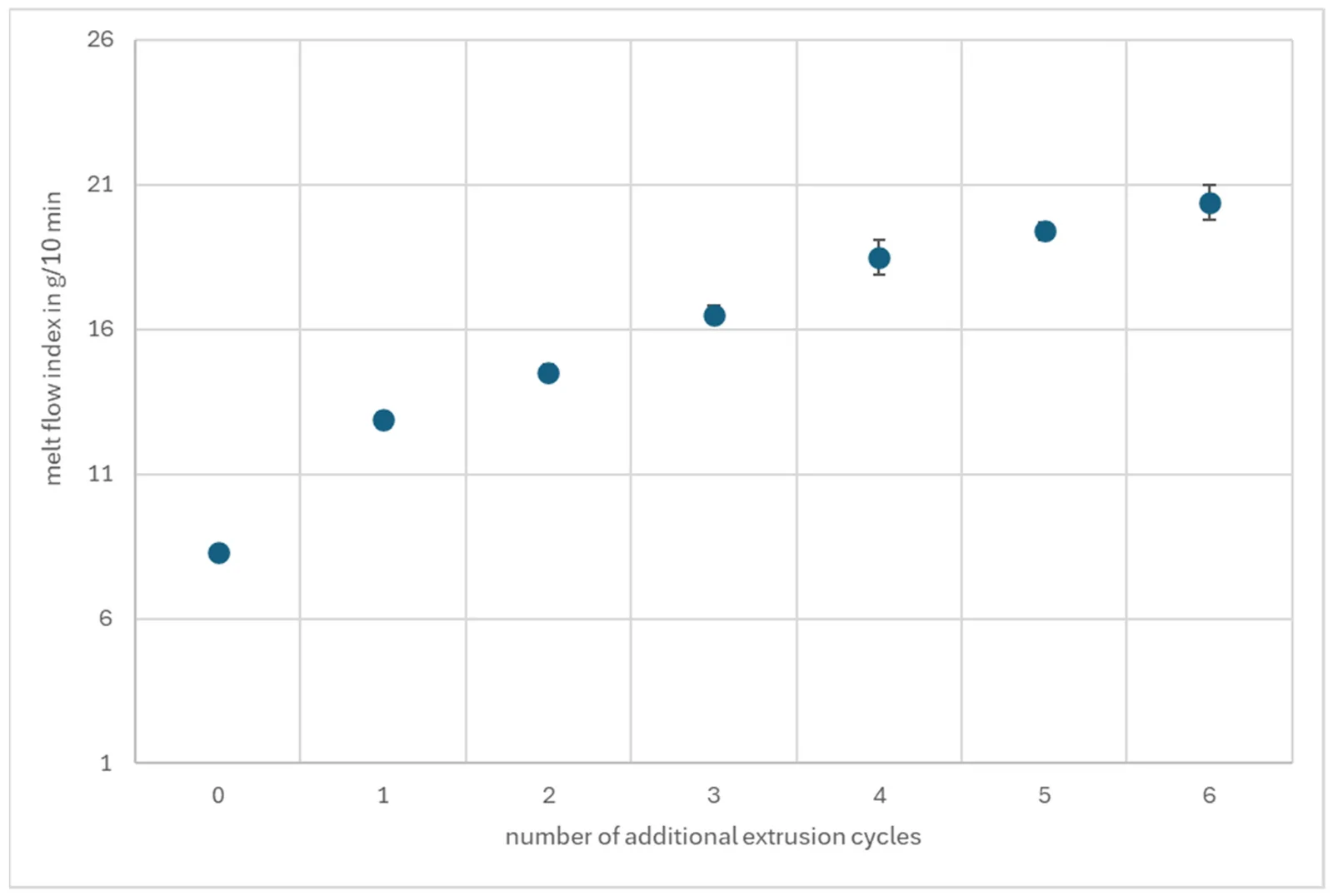
Melt flow index of compound P0 versus number of additional extrusion cycles.

The melt flow index measurements of specimens (granules) from the multiple extrusion cycles show a monotonic increase from 8.3 g/10 min to 20.4 g/10 min with every further extrusion cycle, as shown in Figure 8. This can be attributed to a reduction in the molecular weight of polypropylene due to thermal degradation with each extrusion cycle [39,40,41] and the increase in the melt flow index with decreasing molecular weight [42,43]. However, a decrease in molecular weight is typically associated with fewer tie molecules and entanglements and hence a reduced impact strength [42]. Since the impact strength was found to increase with the number of extrusion cycles over three cycles, this can only be attributed to better bonding at the fiber–matrix interface.

**Figure 9:**
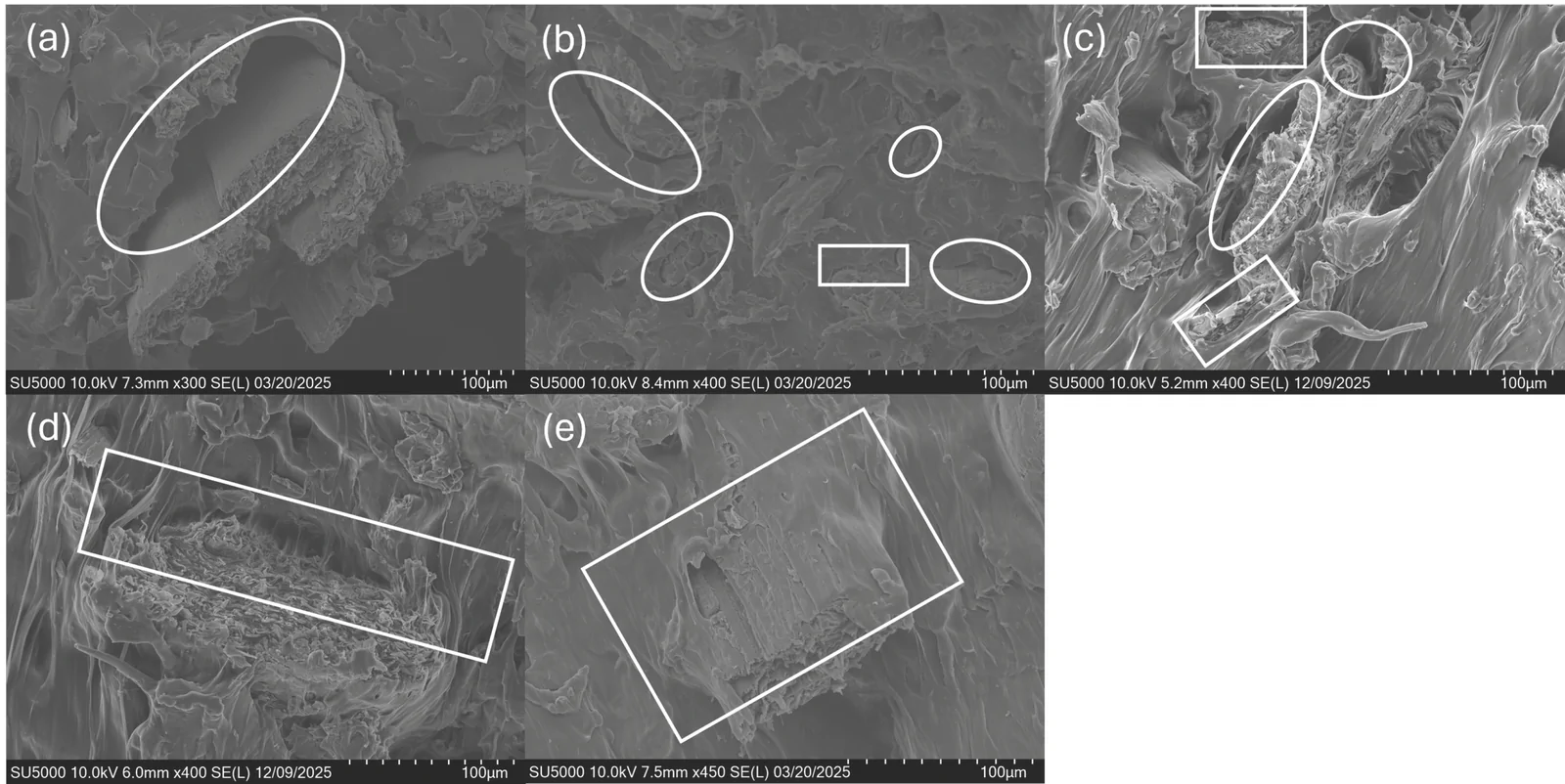
SEM images of front surfaces of cut granules from compound P0 and compounds P1–4 revealing gaps (ellipsoids) and adhesion (squares); (**a**) P0: gaps visible, no adhesion between matrix and fibers; (**b**) P1: mainly gaps, but also adhesion between fiber and matrix visible; (**c**) P2: some gaps, more adhesion between matrix and fiber visible; (**d**) P3: mainly adhesion between matrix and fiber visible; (**e**) P4: mainly adhesion between matrix and fiber visible.

SEM examinations were carried out to better assess the mixing and adhesion between fibers and the matrix. Figure 9 shows the front surface of granules just after the granulation process. No further cutting or breaking was performed beyond that during granulation. The compound shown in Figure 9a does not contain any bonding agent. In this case, distinct gaps between the fiber and the matrix are visible, and no adhesion is observed. The clearly defined gaps serve as starting points for crack propagation.

In Figure 9b–e, the bonding agent content is increased from 1.5% to 7%. As the bonding agent concentration increases, less pronounced gaps become visible and the so-called pull-out effects decrease. Hence, the adhesion between the matrix and the fiber apparently increases as well. At a bonding agent concentration of 5% or higher, no gaps can be detected. Therefore, the adhesion between the matrix and the fiber is dominant at these concentrations.

The SEM images shown confirm the assumption that increasing the amount of bonding agent leads to a better bond between the fiber and the matrix.

**Figure 10:**
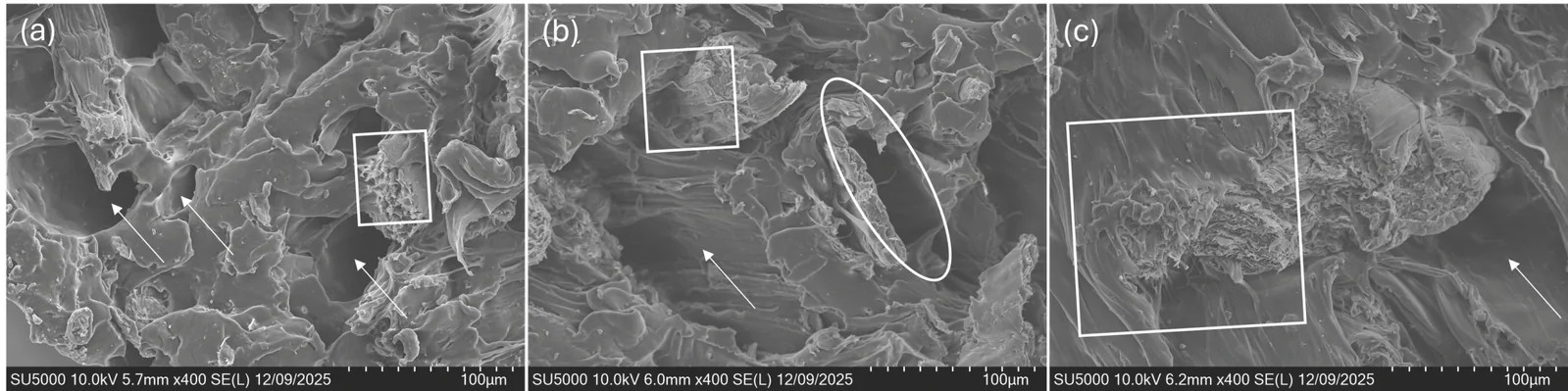
SEM images of front surfaces of cut granules from compounds P11–13 (regrind) revealing gaps (ellipsoids) and adhesion (squares); (**a**) P11: several holes caused by pull-out (marked with arrows) and adhesion between fiber and matrix visible; (**b**) P12: holes caused by pull-out, gaps and adhesion between fiber and matrix are visible (**c**) P13: fewer holes and gaps are visible, adhesion between fibers and matrix is visible.

Figure 10 shows compounds with added impact modifier. While Figure 10a,b show compounds P11 and P12 with only the impact modifier, Figure 10c shows compound P13 with the impact modifier plus the bonding agent. In Figure 10a,b, both adhesion between the fiber and the matrix and distinct gaps are visible. In addition, cavities are visible where fibers were located prior to the granulation process, i.e., pull-out effects are observable, where fibers were pulled out of the matrix by the granulator’s cutting blade due to insufficient adhesion.

It can therefore be concluded that although the impact modifier contributes to a limited extent to the bonding of the fibers to the matrix, this effect is not sufficient to have a significant influence on the impact strength. Rather, the gaps between the fibers and the matrix continue to have a predominant effect on impact strength.

**Figure 11:**
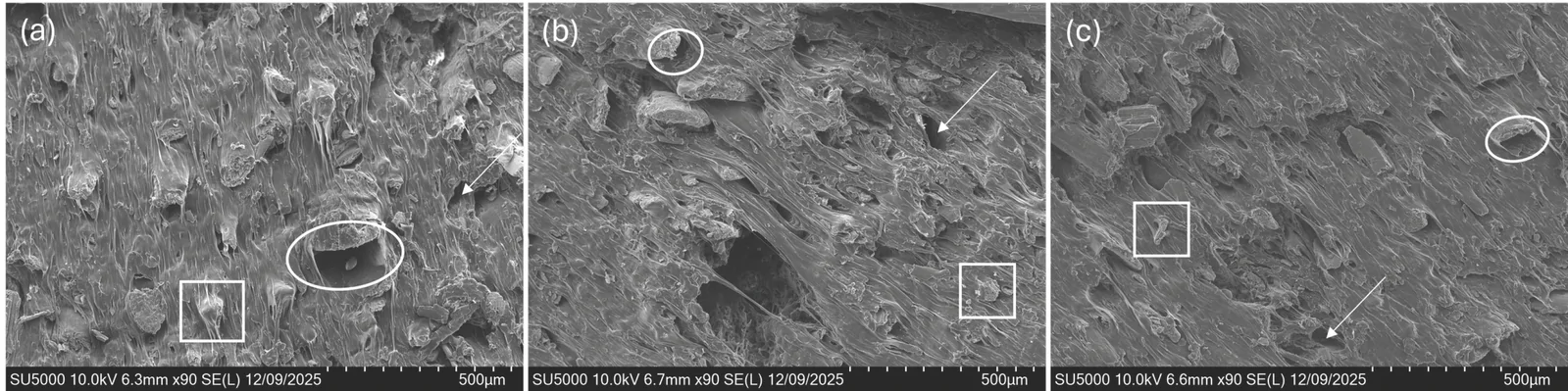
SEM images of surfaces of cut granules from compounds undergoing three different extrusion cycles: (**a**) 2 extrusion cycles, (**b**) 4 extrusion cycles, and (**c**) 6 extrusion cycles. gaps are marked by ellipsoids, adhesion by squares, and holes caused by pull-out with arrows.

Figure 11 shows the front surface of granules after two, four and six extrusion cycles. In all three micrographs, adhesion between fibers and the matrix, gaps and pull-out effects are visible. Figure 11b,c also show a more pronounced covering of the fibers by the polypropylene matrix, which is in line with the findings of the measured impact strength, as shown in Figure 7.

## 4. Conclusions

A virgin polypropylene homopolymer and polypropylene regrind from the same polypropylene were compounded with 30 wt. % grass fibers from permanent pasture grasslands in central Germany. After only one regrinding cycle, no difference in impact strength, tensile strength, and Young’s modulus of the compounds compared to virgin polypropylene was measured.

Three polypropylene-graft-maleic anhydrides with grafting degrees of 1%, 1.4% and 2% were added as coupling agents, as well as EPDM as an impact modifier, to investigate their influence on impact strength. The concentrations ranged from 1.5% to 7% for the coupling agents and 3% and 5% for the impact modifier. The impact strength and melt flow index of the compounds were measured, while the morphology was investigated by scanning electron microscopy.

It was observed that compounds of grass fibers in polypropylene, coupling agents have a larger effect on impact strength than impact modifiers, leading to the conclusion that the interface between fibers and the matrix serves as the predominant factor influencing impact strength. This can be demonstrated by multiple extrusion cycles as a means to reduce the molecular weight of polypropylene and hence increase the melt flow index. The reason for this is because a lower number of tie molecules and entanglements is known to reduce impact strength. However, on the contrary, impact strength increases with the number of extrusion cycles towards a constant level. This can be attributed to stronger bonds across the fiber–matrix interface. Better adhesion is also evident from SEM micrographs, in which it can be seen that the addition of coupling agents reduces gaps and fiber pull-out effects, which cannot be observed when impact modifiers alone are added. Also, specimens from multiple extrusion cycles show better coverage of fibers by the matrix.

## Figures and Tables

**Table 1:** Identification of matrix material, fibers, coupling agents and impact modifier.

Identification	Type of Material	Trade Name	Additional Information
Matrix	polypropylene homopolymer	TOTAL PPH 7062	virgin/regrind
Fibers	grass from permanent pasture grasslands (region Vogelsberg, Germany)	-	-
Coupling agent (C1)	maleic anhydride-g-PP	PA-BOND 363C	degree of grafting 1.0%
Coupling agent (C2)	maleic anhydride-g-PP	SCONA TPP 8112	degree of grafting 1.4%
Coupling agent (C3)	maleic anhydride-g-PP	SCONA TSPP 10112	degree of grafting 2.0%
Impact modifier (IM)	ethylene propylene diene rubber	MELOS EPDM granules	item 462440

**Table 2:** Rheological properties of polypropylene homopolymer and coupling agents according to material data sheets from suppliers according to ISO 1133 or ASTM D-1238.

Material	Melt Flow Index MFI [g/10 min]
virgin polypropylene @ 2.16 kg and 230 °C (ISO)	12
coupling agent (C1) @ 2.16 kg and 230 °C (ASTM)	90–140
coupling agent (C2) @ 2.16 kg and 190 °C (ISO)	>80
coupling agent (C3) @ 1.2 kg and 170 °C (ISO)	40–100

**Table 3:** Width, thickness, width-to-thickness ratio, and length-to-width ratio of grass fibers from three clusters “coarse yellow fibers”, “fine yellow fibers”, and “fine green fibers”.

Classification	Width [µm]	Thickness [µm]	Width-to-Thickness Ratio	Length-to-Width Ratio
coarse yellow fibers	1180 ± 280	89 ± 33	13.3	17
fine yellow fibers	281 ± 102	114 ± 89	2.5	71
fine green fibers	144 ± 38	84 ± 23	1.7	139

**Table 4:** Composition of compounds (in wt. %); all compounds contain 30 wt. % grass fibers.

Compound	Matrix Content	Matrix Origin	Additives
P0	70%	virgin	0%
P0r	70%	regrind	0%
P1	68.5%	virgin	C1: 1.5%
P2	67%	virgin	C1: 3%
P3	65%	virgin	C1: 5%
P4	63%	virgin	C1: 7%
P5	67%	virgin	C2: 3%
P6	65%	virgin	C2: 5%
P7	63%	virgin	C2: 7%
P8	67%	virgin	C3: 3%
P9	65%	virgin	C3: 5%
P10	63%	virgin	C3: 7%
P11	67%	regrind	IM: 3%
P12	65%	regrind	IM: 5%
P13	62%	regrind	C3: 5% + IM: 3%

**Table 5:** Parameters for injection molding of grass fiber polypropylene compounds.

Injection Molding Parameter	Value
nozzle temperature [°C]	200
mold temperature [°C]	15
injection time [s]	2.09
injection speed [cm^3^/s]	14.1
injection pressure [bar]	740
holding pressure [bar]	460
holding time [s]	7.1
cooling time [s]	15

**Table 6:** Mechanical properties of virgin polypropylene and compounds with virgin PP and regrind containing 30 wt. % grass fibers.

Mechanical Property	Virgin PP	Grass + Virgin PP	Grass + PP Regrind
tensile strength [MPa]	34.0 ± 0.2	26.4 ± 0.5	26.8 ± 0.2
Young’s modulus [MPa]	1570 ± 40	2850 ± 60	2770 ± 20
flexural modulus [MPa]	1040 ± 30	2660 ± 30	2580 ± 20
impact strength (unnotched) [kJ/m^2^]	68 ± 2	13 ± 1	13 ± 2
impact strength (notched) [kJ/m^2^]	3.0 ± 0.3	2.5 ± 0.2	2.5 ± 0.4

## Data Availability

The original contributions presented in this study are included in the article. Further inquiries can be directed to the corresponding author.
